# Salt-Sensitive Ileal Microbiota Plays a Role in Atrial Natriuretic Peptide Deficiency-Induced Cardiac Injury

**DOI:** 10.3390/nu14153129

**Published:** 2022-07-29

**Authors:** Siqi Li, Sishuo Chen, Min Nie, Lijing Wen, Bin Zou, Lingyu Zhang, Jingzhou Xie, Hooi-Leng Ser, Learn-Han Lee, Shunyi Wang, Caixia Lin, Janak L. Pathak, Weijie Zhou, Ji Miao, Lijing Wang, Lingyun Zheng

**Affiliations:** 1 School of Life Sciences and Biopharmaceutics, Guangdong Pharmaceutical University, Guangzhou 510006, China; lisiqi25@163.com (S.L.); chensishuo_gd@163.com (S.C.); 18376336367@163.com (L.W.); zhanglingyu1992@163.com (L.Z.); 13618619731@163.com (J.X.); wangshunyi17@163.com (S.W.); lincaix@163.com (C.L.); 2 Guangdong Engineering Research Center of Oral Restoration and Reconstruction, Guangzhou Key Laboratory of Basic and Applied Research of Oral Regenerative Medicine, Affiliated Stomatology Hospital of Guangzhou Medical University, Guangdong 510182, China; minniezou@hotmail.com (M.N.); j.pathak@gzhmu.edu.cn (J.L.P.); 3 State Key Laboratory of Ophthalmology, Zhongshan Ophthalmic Center, Sun Yat-sen University, Guangzhou 510060, China; zoubin1225@outlook.com; 4 Novel Bacteria and Drug Discovery (NBDD) Research Group, Microbiome and Bioresource Research Strength (MBRS), Jeffrey Cheah School of Medicine and Health Sciences, Monash University Malaysia, Bandar Sunway 47500, Selangor, Malaysia; hooileng_ser@y7mail.com (H.-L.S.); lee.learn.han@monash.edu (L.-H.L.); 5 Department of General Surgery & Guangdong Provincial Key Laboratory of Precision Medicine for Gastrointestinal Tumor, Nanfang Hospital, First Clinical Medical School, Southern Medical University, Guangzhou 510515, China; weijiezhouum@163.com; 6 Division of Endocrinology, Boston Children’s Hospital, Boston, MA 02115, USA; 7 Guangdong Province Key Laboratory for Biotechnology Drug Candidates, Guangdong Pharmaceutical University, Guangzhou 510006, China

**Keywords:** ANP deficiency, cardiac injury, salt-sensitive microbiota, ileal microbiota transplantation

## Abstract

Atrial natriuretic peptide (ANP) activity deficiency contributes to salt-sensitive hypertension in humans and mice. However, the role of ileal microbiota in salt sensitivity in ANP deficiency-related cardiac injury has not been investigated yet. This study used ANP^−/−^ mice to analyze the role of the salt-sensitive ileal microbiome on cardiac injury. ANP^−/−^ mice showed an increase in blood pressure (BP), the heart weight/body weight (HW/BW) ratio, and cardiac hypertrophy compared with wild-type (WT) mice. ANP deficiency did not impact the histological structure but reduced occludin expression in the ileum. Antibiotics significantly relieved BP and cardiac hypertrophy in ANP^−/−^ mice. A high-salt diet (HSD) increased BP, the HW/BW ratio, and cardiac hypertrophy/fibrosis in WT and ANP^−/−^ mice, and an HSD treatment in ANP^−/−^ mice exacerbated these cardiac parameters. The HSD markedly decreased muscularis layer thickening, villus length, and numbers of Paneth and goblet cells in the ileum of WT and ANP^−/−^ mice. Furthermore, the HSD increased the level of TLR4 and IL-1β in ANP^−/−^ mice ileum compared with WT mice. Antibiotics reduced the HW/BW ratio, cardiac hypertrophy/fibrosis, and the level of TLR4 and IL-1β in the ileum, and rescued the muscularis layer thickening, villus length, and numbers of Paneth and goblet cells in the ileum of HSD-ANP^−/−^ mice. Importantly, ANP deficiency induced the colonization of *Burkholderiales bacterium YL45*, *Lactobacillus johnsonii*, and *Lactobacillus reuteri* in the ileum on the NSD diet, which was only observed in HSD-induced WT mice but not in WT mice on the NSD. Besides, the HSD significantly enhanced the sum of the percentage of the colonization of *Burkholderiales bacterium YL45*, *Lactobacillus johnsonii*, and *Lactobacillus reuteri* in the ileum of ANP^−/−^ mice. Ileal microbiota transfer (IMT) from ANP^−/−^ mice to healthy C57BL/6J mice drove *Lactobacillus johnsonii* and *Lactobacillus reuteri* colonization in the ileum, which manifested an increase in BP, the HW/BW ratio, cardiac hypertrophy, and ileal pathology compared with IMT from WT mice. The HSD in C57BL/6J mice with IMT from ANP^−/−^ mice drove the colonization of *Burkholderiales bacterium YL45*, *Lactobacillus johnsonii*, and *Lactobacillus reuteri* in the ileum and further exacerbated the cardiac and ileal pathology. Our results suggest that salt-sensitive ileal microbiota is probably related to ANP deficiency-induced cardiac injury.

## 1. Introduction

All hypertensive people are not salt-sensitive and all salt-sensitive people are not hypertensive. However, salt sensitivity is an independent risk factor for cardiovascular (CV) diseases, in addition to the detrimental prognosis conferred by hypertension [1]. Salt sensitivity is specifically common in older adults who have a higher blood pressure (BP) response to a change in salt intake than young adults and normotensive individuals [2,3]. Excessive salt intake, along with higher salt sensitivity, is the key risk factor for hypertension development [4]. Notably, salt sensitivity contributes to the development of left ventricular fibrosis, hypertrophy, and proteinuria [5,6]. Due to salt sensitivity, multiple factors involved in the adaptation of the CV system to a salt load are impaired [7]. Several studies strongly suggest that genetic aberrations leading to defective regulation of natriuresis are the main causative mechanisms of salt sensitivity [8,9,10].

The natriuretic peptide system, including atrial and B-type natriuretic peptides (ANPs and BNPs, respectively), plays a central role in the regulation of water and sodium homeostasis after salt intake [11]. A lack of ANP activity has been reported in several experimental and natural variants of salt-sensitive hypertension [12,13,14]. Epidemiologic studies confirmed that common *NPPA* (the gene encoding ANP) variants rs5065/rs5063 are associated with an increased risk of stroke and a higher prevalence of myocardial infarction in the general population [15,16,17,18]. ANP knockout mice manifest exacerbated left ventricular hypertrophy after a high-salt diet (HSD) treatment [19]. However, the possible causative relationship between ANP deficiency and salt-sensitive cardiovascular injury is still unclear.

A recent study reported that an HSD alters the intestinal microbial composition in a salt-sensitive Dahl rat hypertension model with abundances of seven microbial taxa [20]. Furthermore, gut dysbiosis was detected in animals [21] and humans [22] with hypertension. The causative role of gut dysbiosis in the genesis of hypertension was demonstrated by transferring dysbiotic fecal samples from spontaneously hypertensive rats (SHRs) to normotensive Wistar–Kyoto rats [23]. Notably, the HSD treatment alters the intestinal microbiota–Th17 axis and, consequently, contributes to higher blood pressure [24]. The terminal ileum is associated with the largest mass of lymphoid tissue in the human body, especially the gut-associated lymphoid tissue and Peyer’s patches that are present mainly in the ileum and, together with Paneth cells, increase to a maximum in the distal ileum [25,26,27]; these systems are essential for the maintenance of microbiota-accommodating immune homeostasis [28]. Therefore, hypertension-linked pathological alterations in the gut are mainly observed in the ileum and proximal colon in adult SHRs or an angiotensin II infusion rat model [29].

Nevertheless, most of the studies on the relationships between gut microbiota and hypertension focused only on the fecal (distal colon) microbiota due to the ease of sampling. A study revealed fundamental differences between the microbiota of the distal ileum and colon [30]. For instance, Firmicutes is a common phylum in both fecal and ileal microbiota. However, the species of Firmicutes within these two sites are different [31]. Notably, a recent study demonstrated that transplantation of the ileal contents from sham rats into the organum vasculosum of the lamina terminalis-lesioned rats produces a chronic hypertensive response to deoxycorticosterone acetate-salt treatment, suggesting that ileal microbiota may be involved in the regulation of the development of hypertension [32].

This study hypothesized that ANP deficiency-altered ileal microbiota composition leads to cardiac injury in ANP deficiency. The present study investigated gut pathology and the ileal microbiome composition, and cardiac injury in WT and ANP^−/−^ mice with or without HSD treatment. Moreover, ileal microbiota transplantation (IMT) from ANP^−/−^ mice to healthy C57BL/6J mice was adopted to investigate the role of ANP deficiency and HSD on salt-sensitive ileal microbiota-mediated cardiac fibrosis/hypertrophy. Our results indicate the possible role of ileal colonization of salt-sensitive *Burkholderiales bacterium YL45*, *Lactobacillus johnsonii*, and *Lactobacillus reuteri* in cardiac injury.

## 2. Methods

### 2.1. Animals and Animal Models

8-week-old specific-pathogen-free (SPF) male ANP^−/−^ and WT mice (C57BL/6J background) were bred and maintained in the same room in an animal facility under SPF conditions, at 20–22 °C under a 12 h light/dark diurnal cycle. Based on previous reports, the diets used in the present study included either a normal-salt diet (NSD, containing 0.55% NaCl in a standard chow [19]) or a high-salt diet (HSD, containing 8% NaCl [33,34,35] in a standard chow) obtained from Guangdong Medical Laboratory Animal Center. All diets were irradiated, autoclaved, and replaced weekly to avoid contamination, and all studies were performed according to a protocol approved by the Ethics Committee for Animal Experiments of Guangdong Pharmaceutical University (approval number: gdpulac298064) following the guidelines of Directive 2010/63/EU of the European Parliament on the protection of animals used for scientific purposes. WT and ANP^−/−^ mice were randomly divided into two groups (*n* = 8–10 per group): (1) mice of the NSD group were fed with an NSD for the entire course of the experiment, and (2) mice of the HSD group received an HSD plus autoclaved water ad libitum over four weeks. Mouse colonies of all tested groups were housed in cages in the same room and maintained by the same personnel. Mice were sacrificed by cervical dislocation to harvest the tissues or ileal microbiota.

### 2.2. Noninvasive Blood Pressure Measurements

Systolic blood pressure (SBP) was measured after HSD treatment for 4 weeks by the indirect tail-cuff occlusion method in conscious animals using a BP-2000 series II Blood Pressure Analysis System (Visitech, Visitech Systems, Apex, NC, USA). The mean of six or eight successive measurements was used as the blood pressure estimate. Body weight was measured on the same day.

### 2.3. Antibiotic Treatments

#### 2.3.1. Chronic Combined Broad-Spectrum Antibiotic Treatment (Designated as “Abx” in Figure Legends)

For chronic microbial depletion, a combination of broad-spectrum antibiotics (Abx) in drinking water were used at the following concentrations [36,37]: 1 g/L ampicillin (A0166, Sigma-Aldrich, St Louis, MO, USA), 0.5 g/L vancomycin (V2002, Sigma-Aldrich,), 1 g/L metronidazole (M3761, Sigma-Aldrich,), and 1 g/L neomycin (N6386, Sigma-Aldrich,) in filtered water supplemented with 10 g/L Splenda^®^ to control for the bitter taste of antibiotic solution. Controls were fed filtered drinking water supplemented with 10 g/L Splenda for 4 weeks. All solutions were filtered through a 0.22 μm filter before administration. Water containers were changed once a day to supply fresh antibiotics. Water bottles were monitored to confirm the consumption, and mice were monitored daily for signs of dehydration, which was not detected. During the treatment, body weight was monitored in all animals every week.

#### 2.3.2. Antibiotic Treatment before IMT

Daily oral gavage can prevent dehydration and allow delivery of a precise dose of antibiotics [38]. Therefore, combined broad-spectrum antibiotics (2 mg/mL vancomycin, 4 mg/mL metronidazole, 4 mg/mL ampicillin, and 4 mg/mL neomycin) were dissolved in DPBS and then filtered with a 0.22 μm filter. A gavage volume of 10 mL/kg body weight was delivered with a stainless-steel tube without prior sedation of the mice for 4 consecutive weeks. Controls received a single gavage of 10 mL/kg body weight of sterile DPBS. A fresh antibiotic concoction was mixed every day. The samples of the fecal and ileal contents were collected, and the levels of microbiota DNA were assayed according to the manufacturer’s instructions for the QIAamp PowerFecal Pro DNA kit (Qiagen) to ensure the depletion of most of the intestinal microbiota before the transplantation experiment.

### 2.4. IMT Procedure

For transplantation of the ileal microbiota, age-matched male ANP^−/−^ mice (i.e., donors) were placed in autoclaved cages and allowed to defecate normally. The donor ileal content was collected immediately after donor mice were sacrificed under anesthesia and placed in a sterile vial. The samples were homogenized and passed through a 200 μm sieve to remove food debris. Then, the samples were centrifuged for 1 min at 2000× *g* at 4 °C. Supernatants were transferred to new tubes and centrifuged for 5 min at 15,000× *g* to collect precipitate. Next, 100 mg of precipitate was resuspended in 800 μL of sterile PBS and diluted to a volume of 2.5 mL to transfer 800 μL/mouse to three recipient mice via gastric gavage every other day, immediately after discontinuation of antibiotic treatments. Three weeks after transfer, half of the recipients were switched to an HSD for 4 weeks. All recipients were continuously transplanted with the ileal microbiota from the donors for 7 weeks and the body weight was measured weekly.

C57BL/6J mice (8 weeks, male) were obtained from the vendor facility isolators and used as the recipient mice. Recipient mice were placed in clean cages and treated with antibiotics via gastric gavage every other day for 4 consecutive weeks before the transfer. Notably, recipient mice were transplanted with the ileal microbiota preparations from WT mice, which were used as a control for the transplantation experiment.

### 2.5. Histological Analysis and Immunofluorescence Staining

Paraffin-embedded gastrointestinal tract sections were stained with hematoxylin and eosin (H&E) and Masson’s trichrome for visualization of fibrotic tissue to evaluate the general morphology and collagen formation. The villus length, numbers of the goblet and Paneth cells, and thickness and fibrosis of the tunica muscularis extrema were quantified to assess intestinal pathology. The goblet cell density of the crypts was analyzed in mice using Alcian blue–periodic acid–Schiff reagent (AB-PAS) stains. Neutral mucins were stained with periodic acid–Schiff, and acidic mucins were stained with Alcian blue. The goblet cells were counted in four villi per slide, and the villus height was measured and averaged. For Paneth cell staining, antigen retrieval of the tissue sections with sodium citrate buffer (pH 6.0) was performed in a steam bath for 10 min followed by blocking of nonspecific binding sites with 10% BSA. Then, the slides were incubated overnight at 4 °C with a 1:100 diluted rabbit anti-mouse lysozyme antibody (Abcam, Cambridge, UK). The slides were incubated for 1 h in a dark room with a 1:100 diluted Alexa Fluor 555-labeled goat anti-rabbit IgG antibody (Life Technologies, Waltham, MA, USA). Finally, the slides were mounted using a mounting medium containing DAPI (Beyotime, Shanghai, China), observed under an Olympus BX51 microscope (Olympus, Tokyo, Japan), and imaged by the DP controller software.

### 2.6. Tissue Collection and RT-qPCR Assays

Total RNA was extracted from the ileal epithelium layer using an RNeasy kit (Qiagen, Hilden, Germany) according to the manufacturer’s instructions. RNA concentrations and the 260/280 nm absorbance ratio were determined using a NanoDrop 2000 instrument (Thermo Fisher Scientific, Waltham, MA, USA). cDNA was reverse transcribed using a PrimeScriptTM RT reagent kit (TaKaRa, Tokyo, Japan) according to the manufacturer’s protocols. Quantitative PCR was performed in duplicate using SYBR Premix (TaKaRa, Tokyo, Japan) in a 96-well plate format on a LightCycler (Roche Diagnostic, Mannheim, Germany). The housekeeping gene β-actin was used to normalize the expression levels between the samples, and non-template controls (NTCs) were used as the negative controls. The primers are listed in Supplementary Table S1.

### 2.7. Western Blotting

Protein was extracted from cardiac tissue with 1× RIPA buffer containing 0.1% SDS. Protein (20 µg) was subjected to SDS-PAGE and transferred to polyvinylidene fluoride membranes. Primary antibodies for β-MHC (1:1000, #AF7533, Beyotime, Shanghai, China), fibronectin (1:1000, #ab2413, Abcam), and GAPDH (1:10,000, #2118S, CST). The appropriate secondary antibodies conjugated to horseradish peroxidase (HRP) were from Santa Cruz Biotechnology (1:10,000). Membranes were visualized with SuperSignal West Pico Substrate (Pierce Biotechnologies, Rockford, IL, USA) or Immobilon Western HRP Substrate (Millipore, Billerica, MA, USA). Quantification was performed by using ImageJ software.

### 2.8. Collection of Ileal Content Samples and Microbial DNA Extraction

Due to the limited amount of bacterial DNA extracted from the ileal content of individual mice, the ileal content from 4 mice of each group was collected as a single sample in sterile tubes and stored at −80 °C until microbial DNA extraction and next-generation sequencing. Microbial genomic DNA was isolated from the ileal content using a QIAamp PowerFecal Pro DNA kit (Qiagen, Hilden, Germany) according to the manufacturer’s instructions. Genomic DNA yields were measured using a NanoDrop 2000 spectrophotometer (Thermo Fisher Scientific, Waltham, MA, USA).

### 2.9. Metagenomic Sequencing Analysis

The quality of the raw sequence data was obtained by shotgun sequencing performed by the Berry Genomics company (Beijing, China). Raw reads were checked for quality by FastQC and quality-filtered using Cutadapt (v 1.15) to trim low-quality reads. Then, HISAT2 (v 2.1.0) was aligned to the mouse genome (mm10) and used to obtain clean host sequences. The non-host reads were aligned to our custom microbial genome collection (all genomes from RefSeq complete genome database, including 3658 bacteria, 51 fungi, and 7361 viruses) using Kraken2 and grouped into distinct taxonomic units according to their species-level classification. The ratio of the total mapped reads of each species normalized by the total mapped microbial reads within each sample was used to represent the relative abundance of the species. The differences in bacterial species characterizing the groups were evaluated by using LEfSe [39].

### 2.10. Statistical Analysis

**Figure 1:**
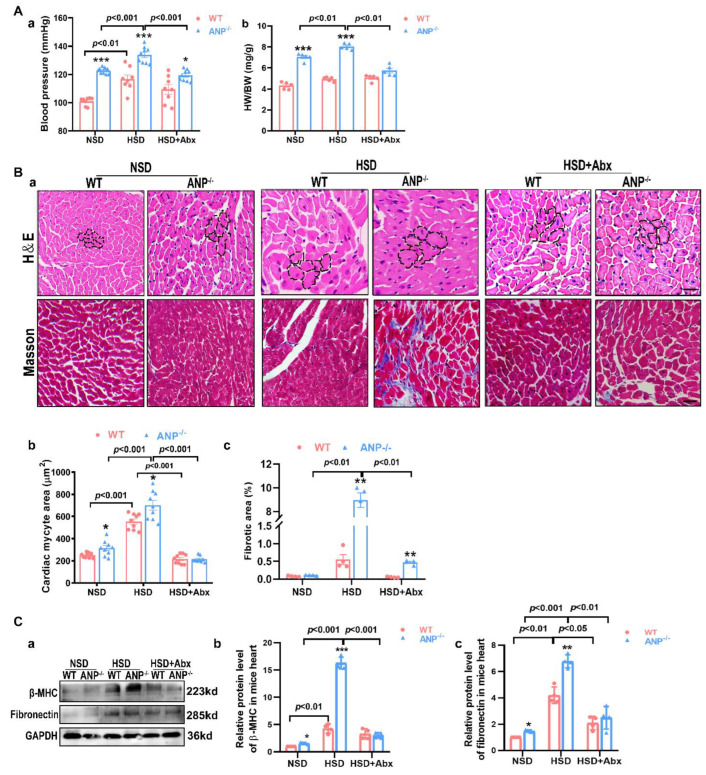
Effects of antibiotics on BP and cardiac pathology in the ANP^−/−^ salt-sensitive model. (**A**) Systolic blood pressure (*n* = 4–7) (**a**), and the heart weight/body weight (HW/BW) ratio (*n* = 4–5) (**b**). (**B**) Representative histological images of H&E- and Masson trichrome-stained cardiac sections and the myocardial area (**a**)**,** quantification of the myocardial area evaluated from H&E-stained tissue sections (*n* = 9) (**b**), and quantification of cardiac fibrosis from Masson trichrome-stained cardiac sections (*n* = 4) (**c**). (**C**) Representative Western blots of β-MHC and fibronectin (**a**) expression in cardiac tissue, relative quantification of β-MHC (**b**)**,** and fibronectin (**c**) expression from Western blot images, (*n* = 3–4). Scale bar = 50 μm. The *p* values were evaluated by two-way ANOVA. Significant difference compared to wild type, * *p* < 0.05, ** *p* < 0.01, and *** *p* < 0.001.

**Figure 2:**
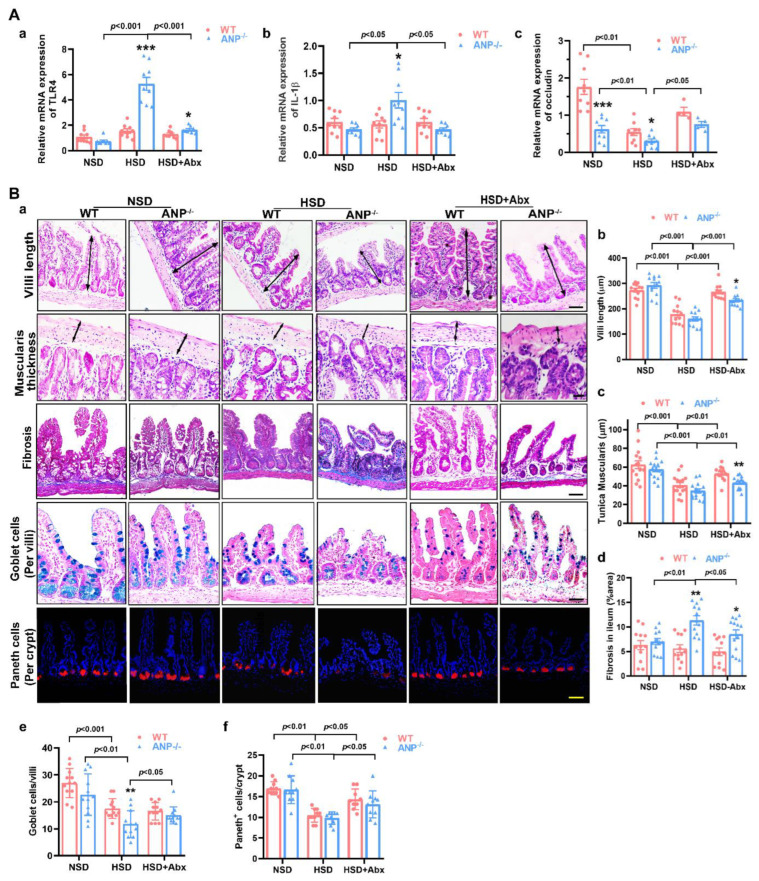
Effects of antibiotics on pathological alterations in the gut of the ANP^−/−^ salt-sensitive model. (**A**) Relative mRNA expression of TLR4 (**a**)**,** IL-1β (**b**), and occludin (**c**) in the ileal epithelium (*n* = 9). (**B**) (**a**) Cross-sections of the ileum of WT and ANP^−/−^ mice stained with hematoxylin and eosin to measure the villus lengths and thickness of the tunica muscularis layer; Masson trichrome staining to quantify the ileal fibrosis area; Alcian blue–periodic acid–Schiff reagent staining to quantify the number of goblet cells; lysozyme (red) staining at the base of the ileal intestinal crypts in mice to quantify the number of Paneth^+^ cells. Quantitative analysis of villi length (**b**), tunica muscularis (**c**), fibrosis in ileum (**d**), goblet cells/villi (**e**), and Paneth^+^ cells/crypt (**f**). Scale bars = 50 μm. The *p* values were evaluated by two-way ANOVA. Significant difference compared to wild type * *p* < 0.05, ** *p* < 0.01, and *** *p* < 0.001 vs. WT.

**Figure 3:**
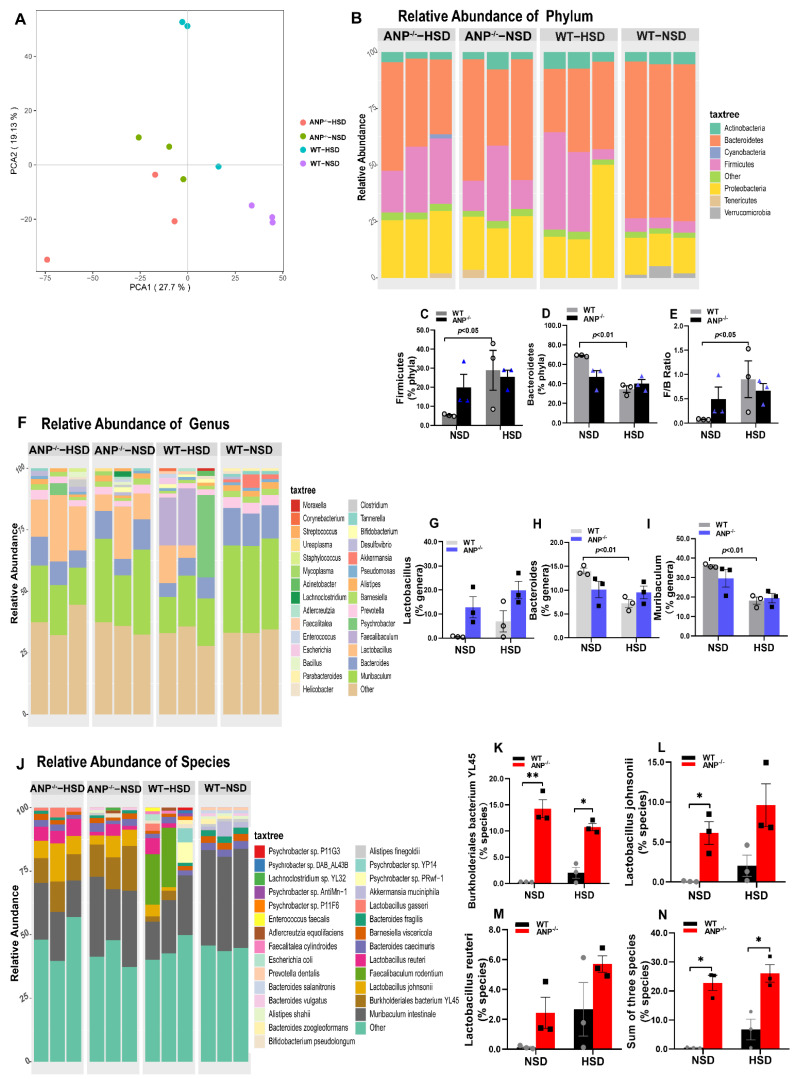
Ileal microbiota features are determined by metagenomic sequencing analysis. (**A**) Principal component analysis (PCA) for species-level ileal microbiome abundance profile. Each symbol represents a sample of ileal microbiota collected from four mice of each group: ANP^−/−^ mice fed an NSD (green points); WT mice fed an NSD (purple points); ANP^−/−^ mice fed an HSD (red points), and WT mice fed an HSD (blue points). (**B**) The relative abundance of individual phyla. (**C**) Percentage of the Firmicutes phylum in total bacteria. (**D**) Percentage of Bacteroidetes phylum in total bacteria. (**E**) The ratio of Firmicutes to Bacteroidetes (F/B). (**F**) The relative abundance at the genus level. Quantification of *Lactobacillus* (**G**), *Bacteroides* (**H**), and *Muribaculum* (**I**) genus level. (**J**) The relative abundance of the microbiome at the species level. The percentages of quantification of *Burkholderiales bacterium YL45* (**K**), *Lactobacillus johnsonii* (**L**), *Lactobacillus reuteri* (**M**), and salt-sensitive species (**N**). Statistical analysis was performed by a one-way ANOVA non-parametric test. Significant difference between the groups, * *p* < 0.05 and ** *p* < 0.01.

Results are presented as the means ± SEM. Statistical differences among treatment groups in Figure 1 and Figure 2 were analyzed by the two-way ANOVA method, followed by Bonferroni’s post hoc test to determine differences between WT and ANP^−/−^ mice in the study parameters. Dunnett’s test was used to determine differences among treatments (NSD, HSD, or HSD + Abx) in the same type of mice, and set the HSD as a common control. For comparison of microbiota percentages among groups in Figure 3, a one-way ANOVA nonparametric test was used. A two-tailed *p*-value < 0.05 was considered statistically significant. All statistical analyses and plotting were performed with Prism software (GraphPad Prism for Windows, version 8.0, San Diego, CA, USA). Differences were considered significant at * *p* < 0.05, ** *p* < 0.01, and *** *p* < 0.001.

## 3. Results

### 3.1. Antibiotics Attenuated HSD-Induced BP and Cardiac Pathology of the ANP^−/−^ Salt-Sensitive Mouse Model

To investigate whether salt-sensitive injury induced by ANP deficiency is linked to the gut microbiota, we administered broad-spectrum antibiotics in drinking water for 4 weeks to deplete most of the intestinal microbiota in ANP^−/−^ and WT mice. Notably, the HSD treatment increased the amount of bacterial DNA in ileal content and feces of ANP^−/−^ mice compared with that of WT mice (Supplementary Figure S1A,B), suggesting a distinct alteration in the ileal microbiota in ANP^−/−^ mice during the HSD. Antibiotics markedly decreased the amount of bacterial DNA in the ileal content and the feces of HSD-treated ANP^−/−^ and WT mice, confirming the effectiveness of antibiotic regiments. ANP knockout enhanced systolic BP compared with that in WT mice (Figure 1A(a)). HSD treatment increased systolic BP in ANP^−/−^ and WT mice. Antibiotics alleviated systolic BP only in ANP^−/−^-HSD mice, but not in WT-HSD mice. ANP^−/−^ mice showed higher HW/BW compared with WT mice (Figure 1A(b)). HSD treatment did not affect the HW/BW in WT mice, but induced HW/BW in ANP^−/−^ mice. Similarly, antibiotic treatment effectively reduced the HSD-induced HW/BW in ANP^−/−^ mice, but not in WT mice (Figure 1A(b)). These results indicate a possible connection between ANP deficiency-induced salt sensitivity and intestinal microbiota with cardiac functions.

Furthermore, we examined cardiac pathological alterations in mice (Figure 1B). HSD treatment caused moderate cardiomyocyte hypertrophy in WT mice (Figure 1B(a,b)), and this effect was nullified by antibiotics (Figure 1B(a,b)), indicating that HSD-induced cardiac pathological changes in WT mice are linked to gut microbiota. In line with other reports, ANP deficiency resulted in enlarged cardiomyocytes but did not impact fibrotic changes compared with WT mice (Figure 1B(a,b)). Notably, antibiotics reduced the BP (Supplementary Figure S2A), HW/BW (Figure S2B), and cardiomyocyte area in ANP^−/−^ mice (Supplementary Figure S2C(a)), suggesting that cardiac hypertrophy in ANP^−/−^ mice was linked to microbiota. Importantly, ANP knockout mice were more vulnerable to the HSD than WT mice and manifested cardiomyocyte hypertrophy (Figure 1B(a,b)) and an increased fibrotic area (Figure 1B(a–c)). Antibiotics reduced the exacerbated cardiac hypertrophy (Figure 1B(b)) and fibrosis (Figure 1B(c)) in HSD-treated ANP^−/−^ mice, further emphasizing the key role of the gut microbiota in the development of salt-sensitive cardiac injuries in ANP^−/−^ mice. Western blot analysis also demonstrated that the HSD treatment increased the expressions of β-MHC and fibronectin in the hearts of WT and ANP^−/−^ mice (Figure 1C(a–c)). HSD-treated ANP^−/−^ mice showed robustly higher expression of β-MHC and fibronectin in the hearts compared with WT mice, suggesting that both HSD and ANP deficiency are involved in cardiac remodeling. Furthermore, antibiotics effectively reduced β-MHC and fibronectin (Figure 1C(a,c)) in ANP^−/−^-HSD mice and also markedly reduced the expression of fibronectin cardiac remodeling.

### 3.2. Antibiotics Attenuated HSD-Induced Alterations in Gut Pathology in the ANP^−/−^ Mice

As mentioned above, hypertension-linked gut pathology was mainly detected in the ileum and proximal colon, which are important sites for immune homeostasis of intestinal microbiota. The expression of TLR4 and IL-1β was increased in ANP^−/−^-HSD mice compared with ANP^−/−^-NSD mice (Figure 2A(a,b)), suggesting that the intestinal epithelium was more susceptible to HSD stimulation in ANP^−/−^ mice. The levels of occludin were lower in ANP^−/−^ mice than in WT mice (Figure 2A(c)), indicating ANP deficiency per se injured the tight junction of the ileum. HSD treatment decreased occludin in both ANP^−/−^ and WT mice (Figure 2A(c)). Notably, antibiotics reduced TLR4 and IL-1β expression in ANP^−/−^-HSD mice. Moreover, occludin expression increased in ANP^−/−^ and WT mice subjected to the HSD after antibiotic treatment. These results indicate that the significant alterations in the intestinal inflammatory environment of the HSD-induced ANP^−/−^ mice are probably related to gut microbiota.

Next, we determined whether similar pathological alterations are also present in the genetic model of induced salt-sensitive hypertension. NSD treatment did not influence the histological structure of the jejunum, duodenum, colon, or rectum in ANP^−/−^ mice (Supplementary Figure S3). Furthermore, the villi length, thickness of tunica muscularis, fibrotic area, and the number of goblet cells or Paneth cells in the ileum of WT mice were similar to those in ANP^−/−^ mice (Figure 2B(a–f)), suggesting ANP deficiency alone does not affect the histological structure of the gut. The length of the villi and the thickness of the muscularis layer in the ileum were considerably decreased after HSD treatment in ANP^−/−^ and WT mice (Figure 2B(a–c)). HSD treatment exacerbated the fibrotic changes in the ileal submucosa in ANP^−/−^ mice (Figure 2B(a,d)), but had no significant effect on these parameters of WT mice (*p* > 0.05 versus WT-NSD). Interestingly, the HSD treatment had only slight effects on the epithelium of the duodenum and jejunum in ANP^−/−^ and WT mice (Supplementary Figure S3), suggesting that the ileum may be more susceptible to an HSD. Goblet cells within the ileal epithelium secrete mucus to cover the epithelium, and Paneth cells are responsible for the production of antimicrobial peptides, which is another defense against the pathogens within the gastrointestinal tract. Additionally, the mucosa (lamina propria) of the ileal region is rich in immune cells and encompasses Peyer’s patches [28]. The results of Alcian blue staining indicated that the number of goblet cells in the villi markedly decreased after HSD treatment in WT and ANP^−/−^ mice (Figure 2B(a,e)). Moreover, the amounts of goblet cells were lower in ANP^−/−^ mice than in WT mice after HSD treatment (Figure 2B(a,e)), and the crypt structure was buried. Paneth cell numbers were assayed using immunofluorescent staining for lysozyme, and the results indicated that the number of lysozyme-positive cells was considerably decreased in HSD-fed WT and ANP^−/−^ mice (Figure 2B(a,f)). Our observations of pathological changes in the ileum strongly indicated that an HSD has a more significant impact on the histological structure in the ileum than ANP deficiency alone. Consequently, antibiotics have no apparent effect on the histological structure of the ileum in ANP^−/−^-NSD mice (Figure S3D(a,b)). Importantly, antibiotics markedly relieved the alterations in the villi length, the thickness of the muscularis layer, and the number of Paneth cells in the ileum of HSD-fed WT and ANP^−/−^ mice (Figure 2B). For ANP^−/−^-HSD mice, antibiotics also significantly rescued the fibrotic changes (Figure 2B(d)) and the number of goblet cells (Figure 2B(e)) in the ileum. Taken together, ileal microbiota might be involved in the alterations of ileal pathology.

### 3.3. The Development of the Salt-Dependent Ileal Microbial Community in ANP^−/−^ Mice

Based on previous results, we assumed that the ileal bacterial taxa are involved in the effect of the HSD or/and ANP deficiency on cardiac and ileal injuries. The distal ileum is an important site for digestion due to the food transit-slowing process and harbors a dense bacterial community (up to 10^8^ bacteria/g). Therefore, we characterized the microbiota composition in the ileum of WT and ANP^−/−^ mice fed an NSD or HSD by metagenome sequencing. An average of 14.8 Gb clean reads was aligned to the reference genomes from the National Center for Biological Information (NCBI) catalog. Principal component analysis (PCA) plots generated using R showed good bacterial type-specific clustering and resolution of the individual read datasets at the species level between the WT-NSD (purple dots) and ANP^−/−^-NSD groups (green dots), and the dispersion of the data points in the WT-HSD (blue dots), ANP^−/−^-NSD (green dots), and ANP^−/−^-HSD (red dots) groups were relatively small, suggesting lower variance and higher similarity of data points in these groups (Figure 3A). Similar to humans, the Actinobacteria, Bacteroidetes, Firmicutes, and Proteobacteria were dominant phyla in the ileum of ANP^−/−^ and WT mice (Figure 3B). Importantly, the results of the phylum-level analysis showed that HSD treatment alone increased the level of the phylum Firmicutes (Figure 3C) and reduced the level of Bacteroidetes in WT mice (Figure 3D). The ratio of the Firmicutes to Bacteroidetes (F/B ratio) is an indicator of gut microbial dysbiosis [6], which was significantly enhanced in the WT-HSD compared with WT-NSD mice (Figure 3E). Notably, this ratio was recently reported to be increased in SHRs and patients with essential hypertension [40]. Therefore, our results suggested that the elevated BP and cardiac pathology induced by an HSD in WT mice were closely associated with the development of gut dysbiosis. Similarly, ANP deficiency per se moderately increased the level of Firmicutes and decreased Bacteroidetes compared to WT mice, but there is no significant difference probably due to the limitation of samples.

The *Lactobacillus* genus (orange cube in the stacked plot, Figure 3F), which belongs to the *Firmicutes* phylum, was detected only in WT mice after HSD treatment, suggesting the *Lactobacillus* genus may be salt-sensitive and salt-dependent. Interestingly, the same genus also was found in ANP^−/−^ mice on an NSD and was moderately enhanced by the HSD treatment (Figure 3G), indicating that ANP deficiency probably induced the *Lactobacillus* genus as well. *Bacteroides* (light blue cube in Figure 3F) were a common genus in all mice and were decreased in WT mice after HSD treatment (Figure 3H), whereas the level of *Bacteroides* genus in ANP^−/−^-NSD mice was not an apparent alteration compared with that in ANP^−/−^-HSD, suggesting that ANP deficiency has no impact on the genus. The level of *Muribaculum* genus (green cube in Figure 3H), which belongs to the *Bacteroidetes* phylum, was markedly reduced after HSD treatment in WT mice (Figure 3I) and moderately decreased in ANP^−/−^ mice (Figure 3I), suggesting the inhibitory effect of the HSD on *Muribaculum*.

Furthermore, the results of bacterial species analysis shown in Figure 3J indicated considerable differences in the composition of the species between the WT-NSD and ANP^−/−^-NSD groups, which were also indicated by PCA results (Figure 3A). Specifically, WT mice treated with an HSD mainly manifested colonization of three species in the ileum, i.e., *Burkholderiales bacterium YL45*, *Lactobacillus johnsonii*, and *Lactobacillus reuteri* (indicated by rose red, dark yellow, and brown cubes, respectively, in the stacked bar chart, Figure 3J). These species were not detected in the WT-NSD group, suggesting these three species are salt-sensitive bacteria. However, there was no significant difference in the abundance of these three species after HSD treatment in ANP^−/−^ and WT mice; this might be due to the small sample size and the variations in HSD intake in individual mice (Figure 3K–M). Interestingly, ANP deficiency promoted colonization of the three species in the ileum of mice treated or untreated with the HSD (Figure 3J). The levels of *Burkholderiales bacterium YL45* and *Lactobacillus johnsonii* were significantly increased in ANP^−/−^ mice compared with WT mice (Figure 3K,L). HSD treatment relatively decreased the level of *Burkholderiales bacterium YL45* in ANP^−/−^ mice (Figure 3K), but moderately enhanced the level of *Lactobacillus johnsonii* (Figure 3L). Similarly, the level of *Lactobacillus reuteri* relatively increased in ANP^−/−^-NSD mice compared with WT-NSD mice. HSD treatment showed an enhancing trend in the level of *Lactobacillus reuteri* in ANP^−/−^ mice (no significant difference, *p* > 0.05, Figure 3M). Considering that bacterial species *Burkholderiales bacterium YL45*, *Lactobacillus johnsonii*, and *Lactobacillus reuteri* are salt-sensitive and salt-induced, we further examined the difference in the sum of percentages of the three species between the groups. Statistical analysis indicated that the sum of the levels of three salt-sensitive species in ANP^−/−^ mice was higher than that in WT mice treat or untreated HSD mice (*p* < 0.05, Figure 3N), further indicating that ANP deficiency promoted the formation and accumulation of three species in the ileum. Overall, these results strongly indicated that *Burkholderiales bacterium YL45*, *Lactobacillus reuteri*, and *Lactobacillus johnsonii* species are probably salt-sensitive. The HSD and ANP deficiency per se may play a new pivotal role in the formation of salt-dependent bacterial species in the ileal microbiota.

### 3.4. IMT from ANP^−/−^ to WT Mice Caused Cardiac Pathology

**Figure 4:**
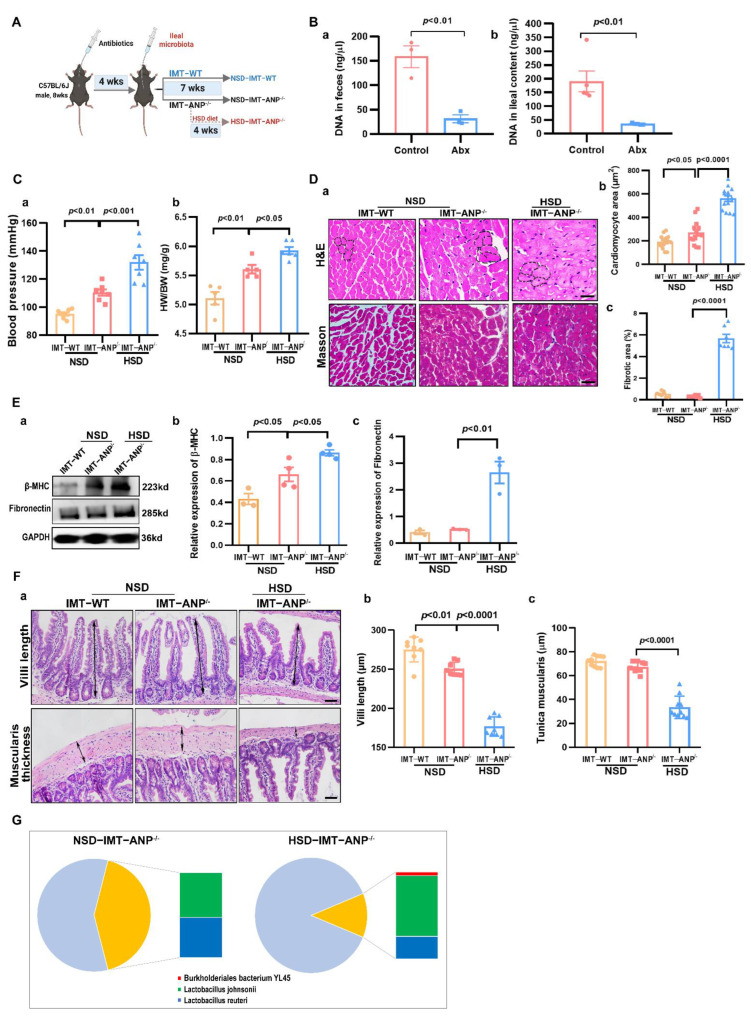
The effect of ileal microbiota transplantation on the cardiac pathology in C57BL/6J mice. (**A**) Schematic representation of the experimental design. (**B**) The amount of bacterial DNA remaining in feces (**a**) and ileal content (**b**). (**C**) Blood pressure (**a**) and HW/BW ratio (**b**) 7 weeks after ileal microbiota transplantation (IMT). (**D**) Heart tissue histology stained with H&E and Masson trichrome (**a**)**.** Quantification of cardiomyocyte area (**b**) and fibrotic area (**c**), *n* = 6. (**E**) Representative Western blots of β-MHC and fibronectin (**a**), relative quantification of β-MHC (**b**)**,** and fibronectin (**c**) expression from Western blot images, (*n* = 3–4). (**F**) Cross-sections of the ileum of recipient mice 7 weeks after the IMT stained with hematoxylin and eosin (**a**). Quantification of villi length (**b**) and tunica muscularis layer thickness (**c**). (**G**) The relative percentage of three salt-sensitive species in recipient mice 7 weeks after the IMT. Scale bar = 50 μM. *p* values were evaluated by one-way ANOVA.

We performed IMT to confirm the role of the microbiota from ANP^−/−^ mice in cardiac pathology. As shown in Figure 4A, the recipient mice were administered antibiotics for 4 weeks by gavage, followed by IMT from WT mice for 7 weeks to obtain NSD-IMT-WT and IMT from ANP^−/−^ mice to obtain the NSD-IMT-ANP^−/−^ group. Three weeks later, some NSD-IMT-ANP^−/−^ mice were fed on the HSD diet for the rest of the 4 weeks to obtain HSD-IMT-ANP^−/−^ mice. Considering the individual variation in drinking water, we used antibiotic treatment for 4 weeks by gavage, and this method significantly removed most bacterial DNA in the feces (Figure 4B(a)) and ileal contents (Figure 4B(b)). IMT-ANP^−/−^ mice showed higher BP and HW/BW compared to IMT-WT mice (Figure 4C(a,b)), and the HSD treatment further enhanced the BP and HW/BW in IMT-ANP^−/−^ mice, suggesting ileal microbiota from ANP^−/−^ mice are responsible for the increased BP and HW/BW in recipient mice. Furthermore, we detected significant left ventricular cardiomyocyte hypertrophy in the IMT-ANP^−/−^ group compared with the IMT-WT group (Figure 4D(a,b)). HSD treatment further exacerbated the extent of cardiomyocyte hypertrophy. Notably, IMT from ANP^−/−^ mice did not result in fibrosis in the heart of recipient mice (Figure 4D(a–c)). Furthermore, the HSD markedly induced fibrotic alterations in the heart of IMT-ANP^−/−^ mice. β-MHC expression was significantly increased in IMT-ANP^−/−^ mice compared with IMT-WT mice (Figure 4E(a,b)), and the HSD enhanced the level of β-MHC in IMT-ANP^−/−^ mice. However, fibronectin expression remained unchanged between IMT-ANP^−/−^ and IMT-WT mice, but the HSD robustly induced fibronectin expression in the cardiac tissue of IMT-ANP^−/−^ (Figure 4E(a,c)). Notably, IMT from ANP^−/−^ mice resulted in the shortening of the villi length in the ileum compared with IMT from WT mice (Figure 4F(a,b)). The HSD further reduced the length of ileal villi in IMT-ANP^−/−^ mice, indicating the synergistic effect of IMT and the HSD on the pathological changes in the ileum. Interestingly, the thickness of the muscularis layer was not altered in IMT-ANP^−/−^ mice compared with IMT-WT mice, but the HSD treatment significantly reduced the thickness of the muscularis layer in IMT-ANP^−/−^ (Figure 4F(c)). Taken together, IMT from ANP^−/−^ mice probably contributed to higher BP, cardiac injuries, and ileal pathology, and the HSD treatment further exacerbated these parameters in IMT-ANP^−/−^ mice.

Therefore, we further analyzed whether salt-sensitive bacteria from ANP^−/−^ mice could be colonized in IMT-recipient mice. Our observations showed that salt-sensitive species including *Lactobacillus reuteri* and *Lactobacillus johnsonii* colonized the ileum of both NSD-IMT-ANP^−/−^ and HSD-IMT-ANP^−/−^ mice (Figure 4G), whereas another species, *Burkholderiales bacterium YL45*, was only colonized in HSD-IMT-ANP^−/−^ mice, indicating that the species could be more susceptible to alterations in the ileal microenvironment and HSD treatment. Notably, three salt-sensitive species could colonize in HSD-treatment-recipient mice. Therefore, BP changes, cardiac injuries, and ileal pathology, which reoccurred in NSD-IMT-ANP^−/−^ and HSD-IMT-ANP^−/−^ mice, probably resulted from ileal microbiota in ANP^−/−^ mice.

## 4. Discussion

Salt sensitivity appears to be a major public health problem, with an estimated incidence of 51% in patients with hypertension and 26% in normotensive people [1]. Salt sensitivity per se is a risk factor for cardiovascular morbidity and mortality [41]. Genetically determined abnormalities in ANP responses to changes in salt balance are the key aberration [19,34,42]. The intestinal tract absorbs nearly all dietary sodium, and the kidneys retain more than 90% of the filtered sodium [43]. During high salt intake, salt-sensitive non-Hispanic blacks manifest a deficiency in ANP secretion and ANP levels did not change among normotensive or salt-resistant subjects [12], suggesting excessive salt absorption might act as a potential contributor to the salt-sensitivity of ANP deficiency. Herein, we found that an HSD alone could result in moderate cardiac hypertrophy and an increase in BP in WT mice after four weeks of treatment, confirming that the effect of HSD-induced salt-sensitive injury likely comes from excessive dietary salt absorption. Expectedly, similar pathological alterations were displayed in ANP^−/−^ mice with a normal salt diet, whereas HSD-induced ANP^−/−^ mice manifested a higher extent of the injury, further demonstrating that salt-sensitive cardiac injury induced by ANP deficiency is closely related to salt absorption. Moreover, our findings demonstrated that microbiota depletion by broad-spectrum antibiotic treatment completely reversed cardiac pathology of the ANP^−/−^ salt-sensitive model, indicating that both the HSD treatment and ANP deficiency-induced salt absorption and cardiac pathology were tightly associated with the intestinal microbiota and immunity. The present study first reported *Burkholderiales bacterium YL45*, *Lactobacillus johnsonii*, and *Lactobacillus reuteri* as salt-sensitive bacterial species in the ileum. IMT from ANP^−/−^ mice induced BP and cardiac pathology in the recipient mice and the HSD further exacerbated these cardiac parameters. Our results indicate the possible role of ileal colonization of salt-sensitive *Burkholderiales bacterium YL45*, *Lactobacillus johnsonii*, and *Lactobacillus reuteri* in cardiac fibrosis/hypertrophy.

Importantly, animal experimental models of hypertension demonstrated the effects of gut microbes on BP [40,44,45,46]. Decreased microbial diversity was detected in animal models of hypertension and human samples [40]. Our observations demonstrated that hypertension-linked gut pathology [29] mainly occurred in the ileum and proximal colon, strongly indicating that the ileal microbiota may be very important for the development of salt-sensitive injury in ANP^−/−^ mice. Results of metagenome sequencing revealed that the composition of the ileal microbiota in ANP^−/−^ mice was altered. HSD alone increased the ratio of Firmicutes to Bacteroidetes and was recently reported to be increased in SHRs and patients with essential hypertension [40], indicating the important role of the HSD in the composition of ileal microbiota. At the genus level, ANP deficiency or HSD alone moderately enriched the content of *Lactobacillus* in the ileum, but the increases are not significant. *Lactobacillus* belongs to the phylum Firmicutes, which are low G+C Gram-positive bacteria generally used as a probiotic for enteric infections [47]; significantly, we found that an HSD induced colonization of *Burkholderiales bacterium YL45*, *Lactobacillus johnsonii*, and *Lactobacillus reuteri* in the ileum of WT mice; these bacteria -were quite low in NSD-WT mice, suggesting the three species are salt-sensitive and salt-induced. ANP deficiency per se promoted the growth of three species in the ileum and, more importantly, *Lactobacillus johnsonii* and *Lactobacillus reuteri* were expended under HSD treatment in ANP^−/−^ mice, strongly suggesting that ANP deficiency probably induced colonization of salt-sensitive bacterial species in the ileum. *Lactobacillus reuteri* are overabundant in obesity [48]. The contents of the order *Lactobacillales* are significantly increased in the coronary artery disease group compared with that of the control group [49], and the *Lactobacillus* genus is enriched in the gut of elderly heart failure patients [50]. Notably, study [24] showed that *Lactobacillus* in feces of FVB/N mice probably were inhibited by a 4% HSD diet and demonstrated that *Lactobacillus murinus* was salt-sensitive and inhibited by NaCl in vitro. Our results showed that in C57BL/6J mice, there was 8% HSD-induced *Lactobacillus* growth in the ileum and, specifically, *Lactobacillus johnsonii* and *Lactobacillus reuteri* were expended by the HSD, which suggested that colonization of *Lactobacillus* in a different segment of the gut exerts a distinct effect and needs a specific microenvironment.

*Burkholderiales bacterium YL45* is an unclassified species that belongs to the unclassified *Burkholderiales* genus. *Burkholderiales* are the Gram-negative bacteria that belong to the phylum Proteobacteria and have been shown to persist in and dominate the gut microbiota of patients with active inflammatory bowel disease (IBD) [51,52,53]. We found that *Burkholderiales bacterium YL45* was slightly induced by an HSD in WT mice, but ANP deficiency alone significantly promoted the species colonization in the ileum. Furthermore, HSD treatment maintained the colonization of *Burkholderiales bacterium YL45* in the ileum of ANP^−/−^ mice and promoted colonization of *Burkholderiales bacterium YL45* in the HSD-treatment-recipient mice, confirming the salt sensitivity of the species.

Similarly, *Lactobacillus johnsonii* and *Lactobacillus reuteri* from ANP^−/−^ mice colonized the ileum of recipient mice treated with or without an HSD. Importantly, these recipient mice manifested cardiac pathology which was observed in ANP^−/−^ mice. Therefore, similar ileal microbiota probably explained the similarity in cardiac injury manifested in ANP^−/−^-NSD, WT-HSD, and IMT-ANP^−/−^ mice. Furthermore, our findings indicated that the HSD treatment has a synergetic impact on the salt-sensitive microbiota in the ileum of ANP^−/−^ mice.

Although broad-spectrum antibiotic treatment for IMT in our study probably results in incomplete ablation of microbes or antibiotic-resistant microbes, many of the effects after antibiotic treatment in mice are consistent with what is seen in germ-free models, such as shifts in cell populations, signaling pathways, and organ morphology, and many phenotype differences after antibiotic treatment that occur are due to broad decreases in bacterial load [54]. Furthermore, using the same batch of WT mice with antibiotic treatment, we observed differential cardiac alterations in mice between those transferred ileal microbiota from ANP^−/−^ and from WT mice. At least, the present study is the first to reveal the features of ileal microbiota in ANP^−/−^ mice and ANP deficiency-induced colonization of salt-sensitive bacterial species in the ileal microbiota. Moreover, salt-sensitive species of *Burkholderiales bacterium YL45*, *Lactobacillus johnsonii*, and *Lactobacillus reuteri* could be colonized in the recipient mice treated with broad-spectrum antibiotics. Significantly, ileal microbiota from ANP^−/−^ mice likely induce BP and cardiac injury, which elucidates a novel origin of salt sensitivity induced by genetic aberration. However, the limitation of the transfer experiment in the present study lacked observation of the HSD-IMT-WT group, considering the quite small percentage of these salt-sensitive microbiota in the HSD-WT group. More work is needed to confirm the effect of IMT on cardiac injury in germ-free mice and prove the salt sensitivity of three strains in the ileum of ANP^−/−^ mice in vitro. Understanding whether salt-sensitive microbiota from ANP^−/−^ mice cause cardiac injury in transfer mice due to Th17-treg imbalance is necessary to investigate in the future.

## Data Availability

The dataset supporting the conclusions of this article is available in the National Center for Biotechnology Information (NCBI) Sequence Read Archive (SRA) repository (BioProject ID PRJNA601633) and can be acquired from NCBI at the following URL: http://www.ncbi.nlm.nih.gov/bioproject/601633 (accessed date 28 February 2021).

## Supplementary Materials

The following supporting information can be downloaded at: https://www.mdpi.com/article/10.3390/nu14153129/s1, Figure S1: The effect of broad-spectrum antibiotics on bacterial DNA amount in ileal content and feces.; Figure S2: Effects of antibiotics on BP, cardiac pathological changes, and ileal pathological alterations in ANP^−/−^ mice; Figure S3: Hematoxylin & Eosin stained histological images of the intestinal tract of WT and ANP^−/−^ mice with NSD or HSD treatment; Table S1: Primers for qPCR of the intestinal epithelium.
