# Supplementary material for: Salt-Sensitive Ileal Microbiota Plays a Role in Atrial Natriuretic Peptide Deficiency-Induced Cardiac Injury
Source: Nutrients. 2022 Jul 29;14(15):3129. doi: 10.3390/nu14153129 (PMC9370783; doi:10.3390/nu14153129)
Supplement: Supplementary file 1 [file nutrients-14-03129-s001.zip › Table S1.pdf]

Table S1. Primers for qPCR of the intestinal epithelium.

Occludin- Forward	ACTCCTCCAATGGCAAAGTG
Occludin- Reverse	CCCCACCTGTCGTGTAGTCT
TLR-4 Forward	CTGGGGAGGCACATCTTCTG
TLR-4 Reverse	CCTCTGCTGTTTGCTCAGGA
IL-1β Forward	GCAACTGTTCCTGAACTCAACT
IL-1β Reverse	ATCTTTTGGGGTCCGTCAACT
β-actin Forward	ATGGTGACGTTGACATCCGTA
β-actin Reverse	GCCAGAGCAGTAATCTCCTTCT
