# Supplementary figures and images for: Salt-Sensitive Ileal Microbiota Plays a Role in Atrial Natriuretic Peptide Deficiency-Induced Cardiac Injury
Source: Nutrients. 2022 Jul 29;14(15):3129. doi: 10.3390/nu14153129 (PMC9370783; doi:10.3390/nu14153129)

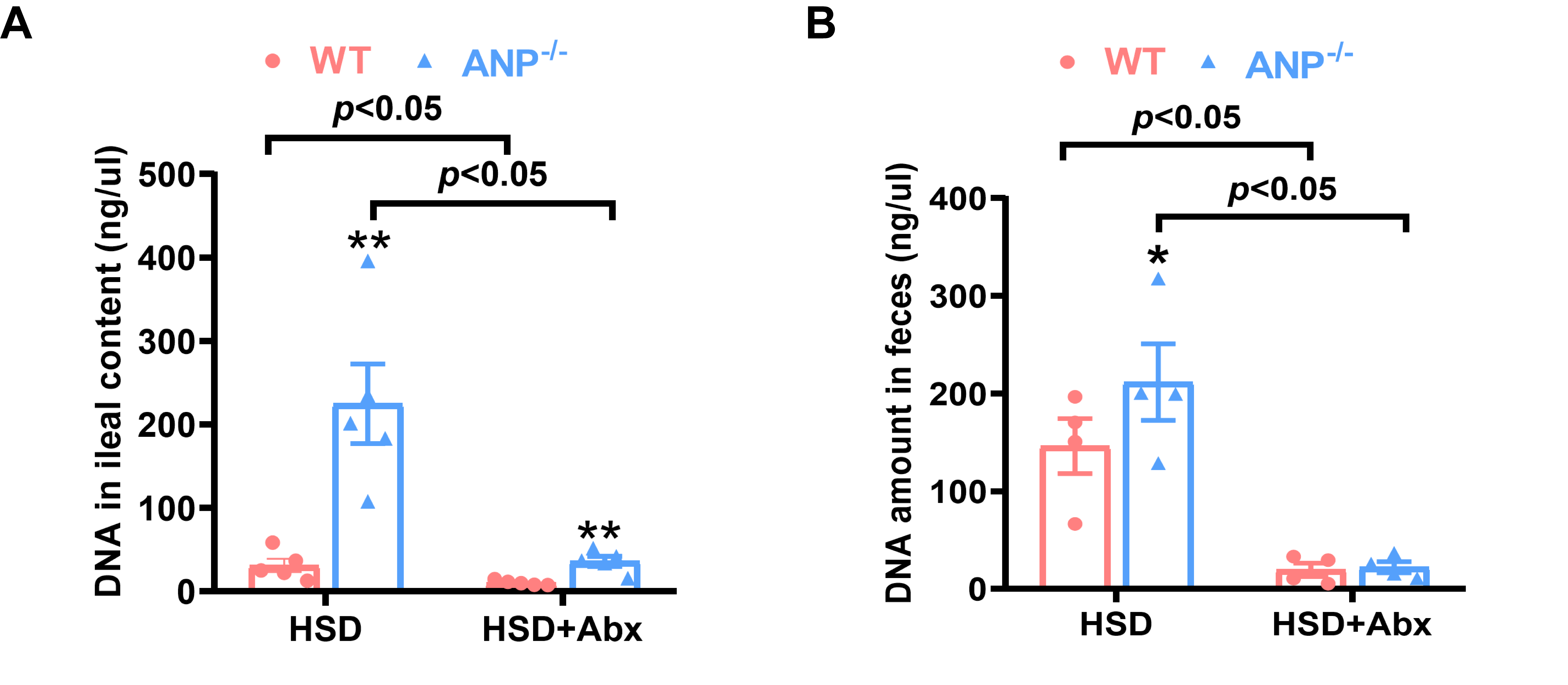

Supplement: Supplementary file 1 [file nutrients-14-03129-s001.zip › supplemental figure S1.tif]

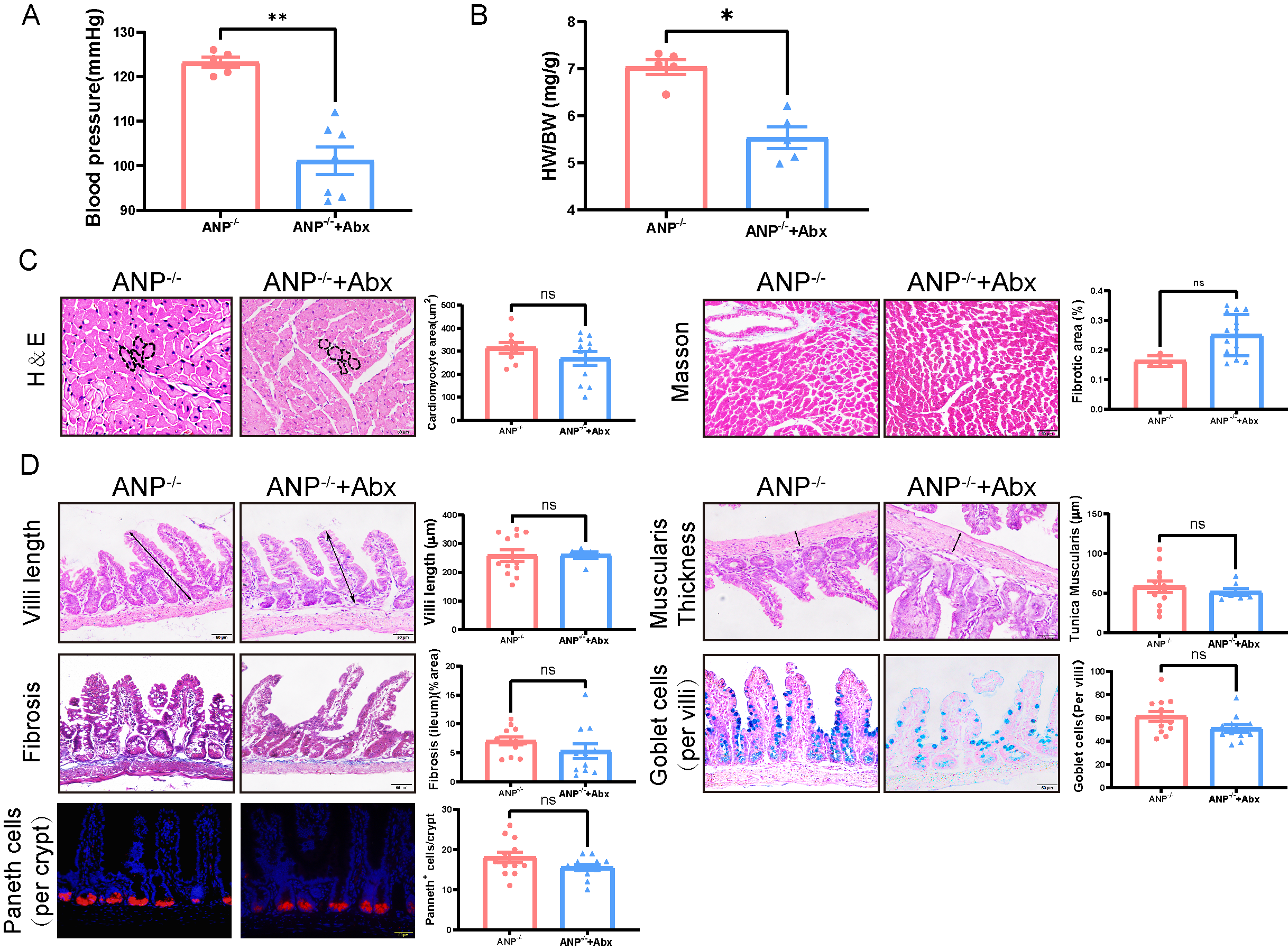

Supplement: Supplementary file 1 [file nutrients-14-03129-s001.zip › supplemental figure S2.tif]
